# The Impact of Visible Symptoms of Thallus Damage on the Phycobiota of Mediterranean Epiphytic Lichens

**DOI:** 10.1007/s00248-026-02794-3

**Published:** 2026-06-09

**Authors:** Tamara Pazos, Patricia Moya, Salvador Chiva, Pavel Škaloud, Veronika Kantnerová, Eva Barreno, Isaac Garrido-Benavent

**Affiliations:** 1 Instituto Cavanilles de Biodiversidad y Biología Evolutiva (ICBiBE) – Botánica, Universitat de València, C/Dr. Moliner 50, Burjassot, València, 46100 Spain; 2 Institut Universitari de Recerca en Biotecnologia i Biomedicina (BIOTECMED), Universitat de València, Av. Vicent Andrés Estellés 19, Burjassot, 46100 Spain; 3 Department of Botany, Faculty of Science, Charles University, Benátská 2, Prague, 12800 Czech Republic; 4 Departament de Botànica i Geologia, Universitat de València, C/Dr. Moliner 50, Burjassot, València, 46100 Spain

**Keywords:** Diversity indices, Illumina, Intermediate Disturbance Hypothesis (IDH), Metabarcoding, Photobiont, Symbiosis

## Abstract

Lichens are excellent bioindicators of overall ecosystem health. The symbiotic nature of their thalli enables tracking changes in humidity, temperature, habitat disturbance and air pollution, often before larger plants do. Sensitive species usually show visible thallus damage, such as bleaching or changes in colour (including total or partial necrosis, and death of the photosynthetic component of the symbiosis), slow growth, and/or biases in reproductive strategies. Particularly, the extent to which these damages are associated with changes in the microscopic photosynthetic community inhabiting lichen thalli (phycobiota) remains poorly understood. Here, we combined Sanger and Illumina sequencing techniques to characterize the diversity and community structure of the eukaryotic phycobiome in selected epiphytic macrolichens showing different levels of thallus damage. Phylogenetic analyses revealed a high microalgal diversity, largely dominated by a few *Trebouxia* species, which are the most prevalent lichenized microalgae, accompanied by several low-abundance co-occurring genera. Notably, microalgal diversity peaked at intermediate levels of thallus damage. This pattern is consistent with disturbance-mediated modulation of microalgal community evenness rather than a categorical shift in symbiotic composition. These findings reveal previously unrecognized variability within the lichen phycobiota, providing new insights into the ecological dynamics and stress responses of these communities. In conclusion, our work offers a new perspective on the potential of lichens as sensitive bioindicators of air quality and ecosystem health.

**Supplementary Information:**

The online version contains supplementary material available at 10.1007/s00248-026-02794-3.

## Introduction

Lichen thalli represent the structural and functional outcome of complex mutualistic interactions between filamentous heterotrophic fungi —referred to as mycobionts— and one or more autotrophic partners, or photobionts, typically green microalgae and/or cyanobacteria [1–3]. Among phycobionts (i.e., lichen-symbiotic green microalgae), most belong to the chlorophyte genus *Trebouxia* Puymaly (e.g. [4–8]). This genus stands as one of the best-studied symbiotic microalgal lineages from taxonomic [9–11], phylogenetic [12, 13], physiological [14–19], genomic [20–22] and geographical perspectives [13, 23, 24]. However, lichenological research has traditionally prioritized the fungal partner, leaving photobiont diversity and evolution comparatively understudied.

Current knowledge of the factors shaping myco-phycobiont interactions is largely based on Sanger sequencing of a single genetic locus [25]. So far, evidence has shown that associations with diverse phycobiont lineages in lichen-forming fungal species can be influenced by both symbiont co-dispersal history [26–29], and environmental conditions, including micro- and macroclimatic gradients [30–33]. At a community level, advances in molecular techniques have propelled the study of myco-photobiont interactions in a variety of ecological contexts, including metal-rich substrata [34, 35], soils [36, 37], rocks [38–40], and forest ecosystems [41–43]. Although these studies have improved our understanding of fungal–photobiont interactions, a new paradigm of lichen symbioses has emerged. Recent evidence revealed that lichens harbor a much greater microalgal diversity than previously recognized [25, 44–46]. These additional algal associates coexist alongside the dominant photobiont in the thallus [45, 47, 48] and from a pure taxonomical perspective, these microalgae are collectively referred to as the phycobiota. Moreover, lichens host diverse bacterial communities (“bacteriota”; [49–53]) and non-lichenized fungi together with the main mycobiont [54–59]. Metagenomic and metatranscriptomic analyses have further confirmed that these microbial communities, along with the mycobiont and the primary photobiont, constitute the lichen holobiome [60–64], reinforcing the view of lichens as self-sustaining microecosystems [3, 65]. The development of high-throughput sequencing technologies, particularly Illumina-based metabarcoding, has brought to light this emerging dimension of lichen biology [66, 67]. These approaches overcome key limitations of Sanger sequencing, which usually detects only the dominant photobiont lineages [25] and may underestimate overall autotrophic diversity within the lichen thallus [38, 44]. When applied complementarily, Sanger and next-generation sequencing (NGS) enable the identification of both predominant and low-abundance microalgal lineages, which might play functional roles in symbiosis and ecological resilience.

As lichen thalli lack protective tissues, they absorb water, nutrients, and gases directly from the atmosphere [68–70] making them particularly sensitive to air pollutants such as sulfur dioxide (SO_2_), nitrogen oxides (NOx), and ammonia (NH_3_), which restrict their distribution and survival [70, 71]. These pollutants have been identified as the primary drivers of lichen decline throughout the past century, reducing the viability of sensitive species and even damaging tolerant ones [72–74]. Changes in lichen diversity in response to environmental stress, including not only air pollution, but also changes in humidity, temperature, forest continuity and other habitat disturbances, make lichens unique, fast indicators of environmental quality [68, 75–80]. Beyond restricting their distribution, these stressors induce visible morphological and physiological damage to lichen thalli, including colour changes, structural degradation, fluctuations in growth rates, and biases in reproductive strategies [81–84]. These observable changes provide a direct, field-applicable link between ecosystem and lichen health [81, 83, 85]. For example, in a recent study from the eastern Iberian Peninsula, Moya et al. [39] assessed the long-term effects of a thermal power plant on epiphytic macrolichen (i.e., with large, easily visible thalli growing on trees) communities in Mediterranean forests. By comparing data from 1997—when the plant was operational—to 2022, two years after its closure, they quantified both species diversity and visible morphological thallus damage using the Damage Index (DI; [86, 87]). Results showed a significant increase in DI at five of seven studied sites, indicating a progressive decline in lichen thallus health. This decline was attributed to the combined effects of atmospheric pollutants, Mediterranean winds transporting contaminants inland, and land-use changes.

Despite growing evidence linking pollution to lichen community shifts, no studies have explored how visible thallus damage relates to the lichen phycobiota. Here, we investigated whether visible morphological damage correlates with changes in the identity of the primary photobiont (*Trebouxia*), and/or shifts in the diversity and composition of accessory microalgae. We explored myco-photobiont associations in a selected community of epiphytic lichens in the same Mediterranean forests studied by Moya et al. [39], combining Sanger sequencing and Illumina-based metabarcoding approaches. Additionally, we tested whether phycobiota’s alpha and beta diversity patterns followed predictions of the Intermediate Disturbance Hypothesis (IDH). This model predicts that species diversity peaks when disturbances are intermediate on the scales of frequency and intensity [88], where competitive exclusion is reduced but communities are not frequently reset by several perturbations [89, 90]. In this context, moderate disturbance can promote coexistence by creating opportunities for colonization and turnover in community composition. Through this framework, we aim to gain new insights into the dynamics of lichen-inhabiting microalgal communities and their responses to environmental stressors.

## Materials and Methods

### Lichen Sampling and Calculation of the Lichen Damage Index

**Fig. 1:**
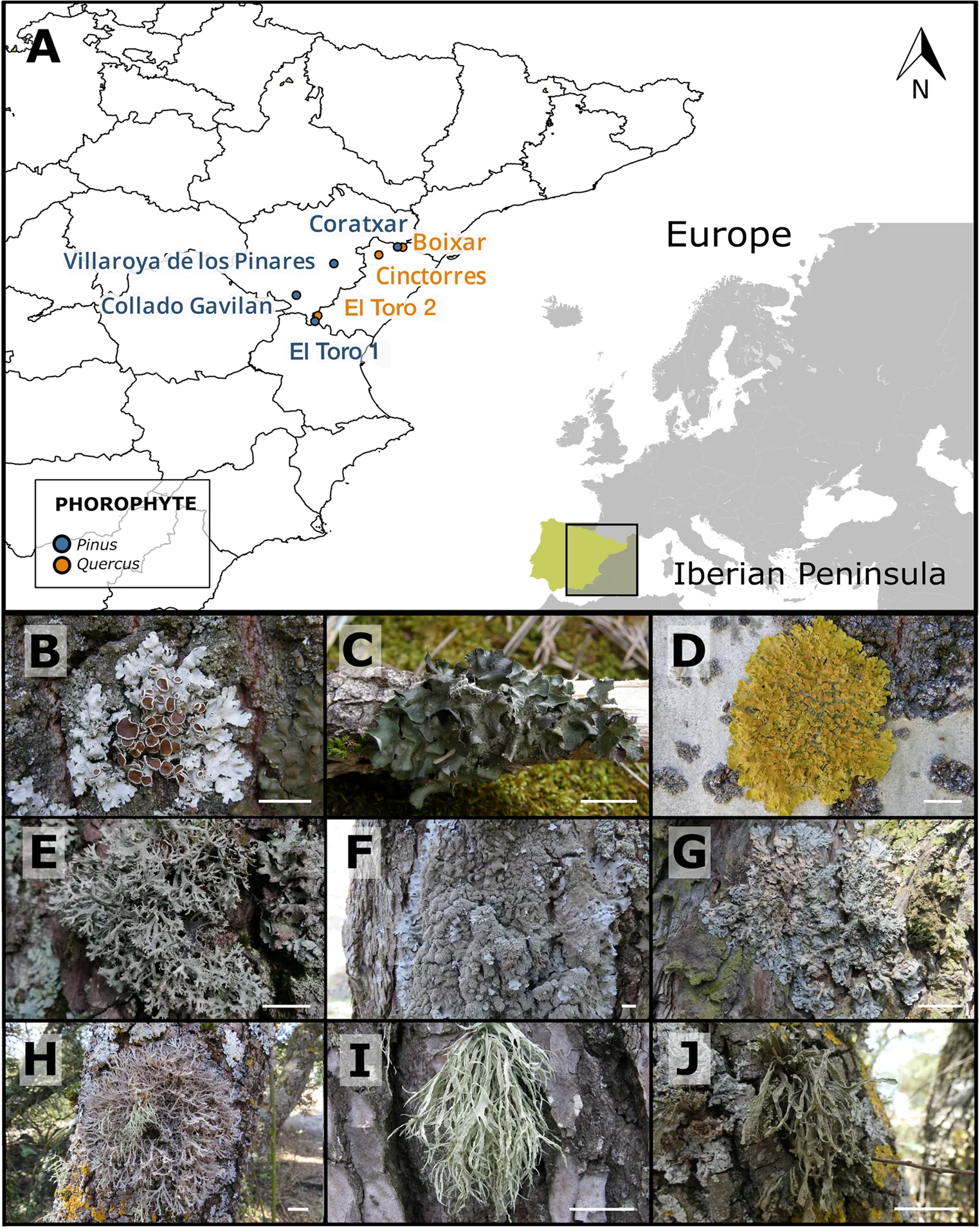
Location of sampling sites in the eastern Iberian Peninsula and details of selected lichen taxa. A, map showing the geographical distribution of visited localities. Boundaries within the Iberian Peninsula correspond with Spanish administrative units. Circle colours indicate the dominant phorophyte in each site: blue—*Pinus* spp. and orange —*Quercus rotundifolia*, B, *Parmelina carporrhizans*, C, *Pleurosticta acetabulum*, D, *Xanthoria parietina*, E, *Pseudevernia furfuracea*, F, *Parmelia serrana*, G, *Physcia aipolia*, H, *Anaptychia ciliaris*, I, *Ramalina farinacea*, and J. *R. fraxinea*. The map was designed using the QGIS software (QGIS Development Team, 2023). Scale bars = 2 cm. Photos: IGB, PM & SC. Figure: TP

We analysed 187 thalli of nine epiphytic lichens collected in 2022 belonging to four fungal families: *Parmeliaceae* (*Parmelia serrana*, *Parmelina carporrhizans*, *Pleurosticta acetabulum*, and *Pseudevernia furfuracea*); *Physciaceae* (*Anaptychia ciliaris* and *Physcia aipolia*); *Ramalinaceae* (*Ramalina farinacea* and *R. fraxinea*); *Teloschistaceae* (*Xanthoria parietina*). Species selection and sampling methodology followed Moya et al. [39], targeting the most frequent and abundant bark-dwelling lichens on the dominant phorophytes in the studied area of the eastern Iberian Peninsula (Spain, south-western Europe): *Pinus* spp. and holm-oak (*Quercus rotundifolia*). Four localities were dominated by forests of *Pinus sylvestris* or *P. nigra* subsp. *salzmannii* and three by holm-oaks (Fig. 1, Supplementary Table S1). At each locality, we aimed at sampling 10 thalli per species. The DI quantification followed the semi-quantitative protocol developed by Barreno et al. [86], and later applied by Moya et al. [39], which recorded visible symptoms of thallus damage (e.g., discoloration, necrosis, twisting —understood as spiraling deformation of thallus lobes or branches—, etc.), their location on the lichen thallus, and the percentage of affected surface. To reduce subjectivity, symptom scoring was standardized and DI values for each lichen were computed based on the number of thalli, their size, and damage intensity on a 1–5 scale (1 = no visible damage; 5 = ~ 90% necrosis or dead thallus; Supplementary Table S1, Supplementary Figure S1). As not all species occurred at every site, thalli per species ranged from 15 (*Parmelina carporrhizans*) to 52 (*Pleurosticta acetabulum*). Samples were stored in Eppendorf tubes with a Nucleic Acid Preservation (NAP) buffer at 4 °C to prevent DNA degradation [91] and preserve lichen present microalgae.

### DNA Extraction, Primary Photobiont PCR Amplification and Sanger Sequencing

Thalli fragments were randomly excised from different parts of each thallus and pooled together. Total genomic DNA was isolated using the DNeasy Plant Mini Kit (Qiagen, Hilden, Germany) following the manufacturer’s instructions. The nuclear ribosomal Internal Transcribed Spacer (ITS) region was selected as the barcode to identify the primary photobiont [6, 13] and to corroborate mycobiont identification based on morphological characters. ITS amplification was done using the primer pairs nr-SSU-1780/ITS4T [92, 93] and ITS1F/ITS4a [94, 95] for microalgae and fungi, respectively. PCR conditions, gel electrophoresis, PCR product purification and Sanger sequencing were done following Moya et al. [96]. Sequence edition and consensus assembly of forward and reverse reads were performed using SnapGene Viewer (v7.0.2) (GSL Biotech. 2023). GenBank accession numbers for all the new sequences are provided in Table S1.

### Assembly of Sanger Sequence Datasets, Alignment, and Myco- and Primary Photobiont Phylogenetic Analyses

The online BLAST search tool [97] was used to check the taxonomic identity at the genus level for all obtained photobiont ITS sequences, considering the GenBank nucleotide database (https://www.ncbi.nlm.nih.gov/genbank/; last accessed 17 November 2025). As all hits belonged to the chlorophyte genus *Trebouxia*, we followed recent phylogenetic assessments of this genus [6, 13, 98] to assemble a phycobiont ITS sequence dataset. We built three datasets encompassing lineages of the three major *Trebouxia* clades A, S, and I [13, 99]. These studies codified each new or undescribed phylogenetic lineage with a capital letter that represented the name of the *Trebouxia* clade and a two-digit numeral, except for clade I, for which we followed the nomenclature proposed by Leavitt et al. [13]. At least one representative sequence for each previously reported species-level lineage (https://trebouxia.net; last accessed 22 November 2025) was downloaded from the GenBank for a total of 64 (clade A), 54 (clade S) and 62 (clade I) ITS sequences. For rooting, *T. lynniae* (A39, GenBank accession number FJ418565) and *T. jamesii* (A03, FJ626733) were included in the clade S dataset, two sequences of *T. simplex* (S10, KJ623927, KJ623934) in clade A, and one sequence of *T. simplex* (S10, FJ626735) plus *Trebouxia* sp. (S21, JN204811) in clade I. For the mycobiont, the ITS dataset combined all the new sequences with the top three BLAST hits (including the type sequence when available). A sequence of *Rhizoplaca melanophthalma* (DC) Leuckert (NR_120221), belonging to the family *Lecanoraceae*, was used as the outgroup.

Alignments were built using MAFFT (v7.505) [100, 101] and the FFT-NS-I x1000 algorithm, the 200PAM/k = 2 scoring matrix, a gap open penalty of 1.5, and an offset value of 0.123. Resulting alignments were manually edited to trim ends of longer sequences and to annotate partitions within the whole ITS region. Phylogenetic inferences were then made following Maximum Likelihood (ML) and Bayesian Inference (BI). ML tree estimations were carried out with RAxML [102, 103] as implemented in the CIPRES Science Gateway [104], using the GTRGAMMA as the nucleotide substitution model for the two defined partitions (ITS1 + 2, 5.8 S). One thousand rapid bootstrap pseudoreplicates were conducted to evaluate nodal support. For BI, PartitionFinder (v1.1.1) [105] was first used to find the optimal evolutionary nucleotide substitution and partition models (Table S2). Then, the BI phylogenetic reconstruction was conducted with the program MrBayes (v3.2.7) [106], with two parallel, simultaneous four-chain runs executed over 5 × 10^7^ generations, starting with a random tree, and sampling after every 500th step. The first 25% of data was discarded as burn-in, and the 50% majority-rule consensus tree and corresponding posterior probabilities (PP) were calculated from the remaining trees. Chain convergence was assessed to ensure that the potential scale reduction factor (PSRF) approached 1.00 and that the average standard deviation of split frequencies (ASDSF) values fell below 0.005. Tree nodes with bootstrap support (BS) values higher than 70% and PP equal or higher than 0.95 were regarded as significantly supported. Trees were visualized in FigTree (v1.4.4) (http://tree.bio.ed.ac.uk/software/figtree/) and artwork finalized in InkScape (v1.3) (https://inkscape.org).

### Interaction Networks and Damage Index of the Studied Lichens

Interaction networks were built using the function plotweb in the R package *bipartite* [107], considering phylogenetically circumscribed phycobiont and mycobiont lineages. Additionally, the registered DI was included to visually explore whether specific microalgal lineages were more frequently associated with either healthy or damaged lichen thalli. To minimize sampling bias and enhance visual clarity, all figures are presented in two categories: healthy lichens (DI = 1, *n* = 70) and damaged (including lichens with DI ranging from 2 to 5, *n* = 83). We quantified interaction specialization using the *d′* index [108] via dfun, testing whether thallus damage influenced specialization patterns. Differences in *d′* among damage levels were evaluated with a Kruskal–Wallis test (kruskal.test; α = 0.05).

### Illumina Assay

Illumina sequencing was performed on 186 lichen samples to explore phycobiota taxonomic composition. This study employed DNA extractions previously used in the Sanger sequencing analysis of the primary lichen photobiont. The DNA concentration of each sample was determined to calculate the total DNA volume to be added to each PCR reaction through a Qubit dsDNA HS assay (Thermo Fisher Scientific, MA, United States) following the manufacturer’s instructions. PCR amplifications of the internal transcribed spacer 2 (ITS2) region were then carried out using the primer pair ITS-Cha3 (5’-CAACTCTCRRCAACGGATA-3’) and ITS U4 (5’-RGTTTCTTTTCCTCCGCTTA-3’ [109]). PCR reactions contained 10 µl of Q5 High-Fidelity DNA polymerase (BioLabs Inc.), 1 µl of each primer, a variable amount of DNA ranging from 0.5 to 7 µl, and sterile water up to a total volume of 20 µl. Reactions for all samples were run in duplicate. Additionally, we included 12 PCR negative controls to check for possible DNA contaminations, hereafter referred to as PNC (i.e., reactions without DNA template), and 24 empty wells, also known as ‘Multiplex Controls’ (MPC, i.e., wells containing no DNA, primers, or polymerase). MPCs were used to detect potential primer jumps during the next-generation sequencing assay, a phenomenon that can occur after pooling of amplicons that carry distinct tags. In these conditions, incompletely extended PCR products or residual tagged primers may transiently anneal to amplicons originated from different samples. During subsequent amplification cycles, these heterologous molecules can be extended by DNA polymerase, resulting in chimeric amplicons. Such chimeric molecules may be interpreted as genuine sequences during demultiplexing, leading to potential overestimation of species diversity [110]. PCR conditions were as follows: initial denaturation at 98 °C for 30 s, 35 cycles of 98 °C denaturation for 10 s, 52 °C amplification for 45 s and 72 °C elongation for 1 min, with a final 72 °C extension for 2 min. After purification with SPRI AMPure XP paramagnetic beads (Beckman Coulter), PCR products were pooled equimolarly and sent for library preparation and sequencing to Fasteris (Plan-les-Ouates, Switzerland). To avoid bias and chimera formation from additional PCR during the library creation, libraries were prepared according to the Fasteris MetaFast protocol. Amplicon paired-end sequencing was performed on an Illumina MiSeq sequencer (2 × 300 bp, Illumina Inc., San Diego, CA, United States) using the MiSeq Reagent Kit (v3). The datasets generated for this study were deposited in the NCBI Sequence Archive with BioProject number PRJNA1471648.

### Bioinformatic Analyses of Sequence Reads and Phycobiota Taxonomic Composition

Quality filtering of Illumina MiSeq paired-end reads was performed using FastQC (v0.11.8) [111], and raw reads processing was performed according to the pipeline published by Báilint et al. [112], subsequently followed by Kantnerová & Škaloud [46]. Read clustering was carried out using Swarm (v2) [113] with a denoising parameter of d = 3, generating denoised amplicons (swarms). Unlike traditional OTU clustering, which applies fixed similarity thresholds (e.g., 97%), Swarm uses an iterative single-linkage algorithm that enables locally adjusted clustering, balancing the broad grouping of OTUs with the fine-scale resolution of ASVs. Swarms were retained for further analysis only if detected in both replicates, present in more than 10 reads, and absent or nearly absent in PNCs and MPCs [114].

For taxonomic assignment, processed microalgal reads, excluding uncultured/environmental samples, were queried against the BLAST database using SEED2 [115] as a preliminary step to retrieve the closest available reference sequences. Only BLAST hits showing > 95% identity were considered for subsequent phylogenetic analyses. An initial global phylogenetic tree was constructed including all microalgal swarms and their corresponding GenBank BLAST hits to delimit genus-level groups based on their phylogenetic clustering.

Based on this preliminary genus-level assignment, the dataset was partitioned by genera, and separate phylogenies were built for each genus to enable species-level identification of the detected swarms using the GenBank sequences retrieved via SEED2. In these genus-specific analyses, the taxonomic assignment of each swarm to the species level was based on its phylogenetic position within the resulting trees. As species-level circumscriptions in several microalgal genera (e.g., *Apatococcus*, *Asterochloris*, and *Diplosphaera*) could not be reliably resolved using similarity thresholds alone, additional databases with GenBank sequences were created following the criteria and methods implemented to build *Trebouxia* phylogenies mentioned above: we generated phylogenetic trees for each genus, including these unassigned swarms together with the GenBank sequences retrieved via SEED2 and sequences representing all formally described species-level lineages for each genus. Based on their clustering patterns within the tree, unidentified swarms were assigned to putative new species. To assess whether these putative species had been previously reported elsewhere, BLAST searches were performed against the NCBI nucleotide database. A sequence of *Interfilum paradoxum* retrieved from the Illumina sequencing was incorporated into the three datasets for rooting purposes, except for *Asterochloris*, where *Trebouxia jamesii* was used for the same purpose. For the microalgal genus *Trebouxia*, the dataset was additionally partitioned into the three major *Trebouxia* clades A, S, and I. Subsequently, phylogenetic analyses were conducted for each clade/genus using RAxML to refine taxonomic identification, as described above.

### Statistical Analyses and Diversity Indices

All community analyses were based on Illumina-derived swarm tables. A phyloseq object was constructed by integrating the taxonomy table of detected swarms, the frequency table, and a metadata table containing the recorded DI for each sample, using the *phyloseq* package [116]. Samples were treated as count data without rarefaction [117, 118]. First, we assessed swarm richness relative to the sequencing depth, calculated as the number of reads per sample, through rarefaction curves generated with the ggrare function from the *ranacapa* package [119] to ensure sufficient sequencing coverage for all samples. Subsequently, alpha diversity was quantified to evaluate whether observed lichen thallus damage influenced the diversity of lichen microalgae. The Shannon Index [120], which measured species richness across DI levels, and the Simpson Index [121], which assessed species dominance across the same levels, were calculated using the estimate_richness function [116]. Differences in alpha diversity among damage levels were tested with the Kruskal-Wallis test, using the function kruskal.test, considering p-values < 0.05 as statistically significant. All analyses were conducted in R (R Core Team 2024) and visualizations were generated with *ggplot2* [122].

The impact of symptoms of thallus damage on the lichen phycobiota was analyzed through non-metric multidimensional scaling (NMDS) based on Bray-Curtis dissimilarity. NMDS ordination was performed using the ordinate function and visualized with plot_ordination [116]. Group variation in NMDS was evaluated using Permutational Multivariate Analysis of Variance (PERMANOVA) with the adonis function from the *vegan* package [123], while within-group dispersion was assessed using the betadisper function and validated with a permutation test with the xi function [123]. To minimize sampling bias and improve visual clarity, all figures are organized into two groups: healthy lichen thalli (DI = 1, *n* = 70) and damaged (including lichens with recorded DI values from 2 to 5, *n* = 83). All diversity analyses were conducted following the workflow described in Hofmann et al. [114].

## Results

### Phyco- and Mycobiont Phylogenetic Analyses of Sanger Sequences

Alignments assembled for *Trebouxia* clades A, I, and S using the newly obtained Sanger ITS data consisted of 167, 84 and 116 sequences, respectively. RAxML analyses estimated topologies with a final LogLikelihood of −6048.5888 (clade A), −4583.6077 (clade I), and − 6490.3082 (clade S). MrBayes analyses reached ASDSF values of 0.005 after 1.55 × 10^7^ (clade A), 5.30 × 10^6^ (clade I), and 4.06 × 10^6^ (clade S) generations. Estimated Sample Sizes (EESs) were above 5000 for all parameters in all cases. As topologies inferred with the two approaches showed no supported conflicts, BI trees are presented in Supplementary Figs. S2-S4. These phylogenies further revealed distinct results of phycobiont diversity across the three *Trebouxia* clades analysed.

#### *Trebouxia* Clade A Harbours High Phycobiont Diversity

The *Trebouxia* clade A phylogeny revealed eight different species-level phycobiont lineages in the nine selected epiphytic macrolichens (Supplementary Fig. S2). Four lineages represented the formally described species *T. decolorans*, *T. jamesii*, *T. lynniae*, and *T. solaris*. *Trebouxia* sp. A50 has been reported previously, but remains undescribed [6]. The remaining three *Trebouxia* are reported in the present study for the first time: *Trebouxia* sp. A68, A69, and A70, following the coding system provided on the *Trebouxia* Research Portal (https://trebouxia.net). These lineages are represented by sequences PP265179, PP265182, and PP265181, respectively. *Trebouxia decolorans* was the most frequent (*n* = 49), occurring in thalli of *Ramalina fraxinea*, *Anaptychia ciliaris*, *Pleurosticta acetabulum*, and *Xanthoria parietina* collected in both *Quercus*- and *Pinus*-dominated forests. The second-most frequent phycobiont was *T. jamesii* (*n* = 28), which was strictly associated with *R. farinacea* and *R. fraxinea* in both forest types. Its sister species, *T. lynniae*, appeared in only two *R. farinacea* thalli located in *Quercus* forests. *Trebouxia solaris* was detected exclusively in a single *X. parietina* thallus in Cinctorres. The two undescribed lineages A68 (*n* = 2) and A70 (*n* = 2) were also only found in lichens collected in Cinctorres associated with *A. ciliaris *and*R. fraxinea*, respectively. Two phycobionts were exclusive to lichens growing on *Quercus*: *Trebouxia* sp. A50 (*n* = 11) in thalli of *A. ciliaris*, *R. fraxinea*, and *X. parietina* from Boixar, Cinctorres, and El Toro 2, and *Trebouxia* sp. A69 (*n* = 8) in *A. ciliaris*, *P. acetabulum*, and *P. carporrhizans* from Cinctorres and El Toro 2.

#### Low Diversity but Strong Dominance of *Trebouxia Barrenoae* in Clade S

In contrast to the relatively high diversity observed in clade A, in *Trebouxia* clade S, only two microalgae partnered with the studied lichenized fungi (Supplementary Fig. S3): *T. simplex* with two *R. farinacea* specimens growing in two localities dominated by *Pinus* spp., and *T. barrenoae* [124], which was the most frequent microalga overall (*n* = 61) and mainly associated with *Pseudevernia furfuracea* and *Parmelia serrana*. *Trebouxia barrenoae* occurred in all *Pinus*-dominated localities, and in El Toro 2 (*Quercus*) where it associated with one thallus of *A. ciliaris* and two *X. parietina* thalli.

#### Previously Unreported *Trebouxia* Lineages Detected in Clade I

Beyond the patterns observed in clades A and S, six species-level lineages in *Trebouxia* clade I were found in lichens growing on *Quercus*-dominated localities (Supplementary Fig. S4): three previously reported, but undescribed (*Trebouxia* sp. I01, I01G, and I01H [13]), and three newly reported here, provisionally named *Trebouxia* sp. I01X, I01Y, and I01Z. *Trebouxia* sp. I01X (*n* = 2) and I01Z (*n* = 2) exclusively associated with *Physcia aipolia*, and *Trebouxia* sp. I01Y (*n* = 3) with *P. aipolia* and *Pleurosticta acetabulum.* The moderately frequent *Trebouxia* sp. I01 (*n* = 10) was found in symbioses with thalli of *P. aipolia* and *Parmelina carporrhizans*. The latter was also involved in symbiosis with *Trebouxia* sp. I01H (*n* = 3). *Trebouxia* sp. I01G (*n* = 2) was found associated with *P. aipolia* and *X. parietina.*

#### Phylogenetic Analyses Corroborate Mycobiont Species Identities

The mycobiont ITS alignment comprised 178 sequences, of which 147 were generated *de novo* and 31 were downloaded from the GenBank. The ML analysis estimated a topology with a final LogLikelihood of −4832.4165, and the MrBayes analysis achieved an ASDSF value of 0.005 after 1.06 × 10^6^ generations. EESs were above 10,000 for all estimated parameters. ML and BI topologies showed no supported conflicts and grouped specimens of the nine studied mycobionts in nine well-supported clades (BS > 95 and PP = 1, Supplementary Fig. S5). Phylogenies corroborated the taxonomic identity of mycobionts, previously assessed based on morphological traits.

### Symbiont Interaction Patterns and the Damage Index

**Fig. 2:**
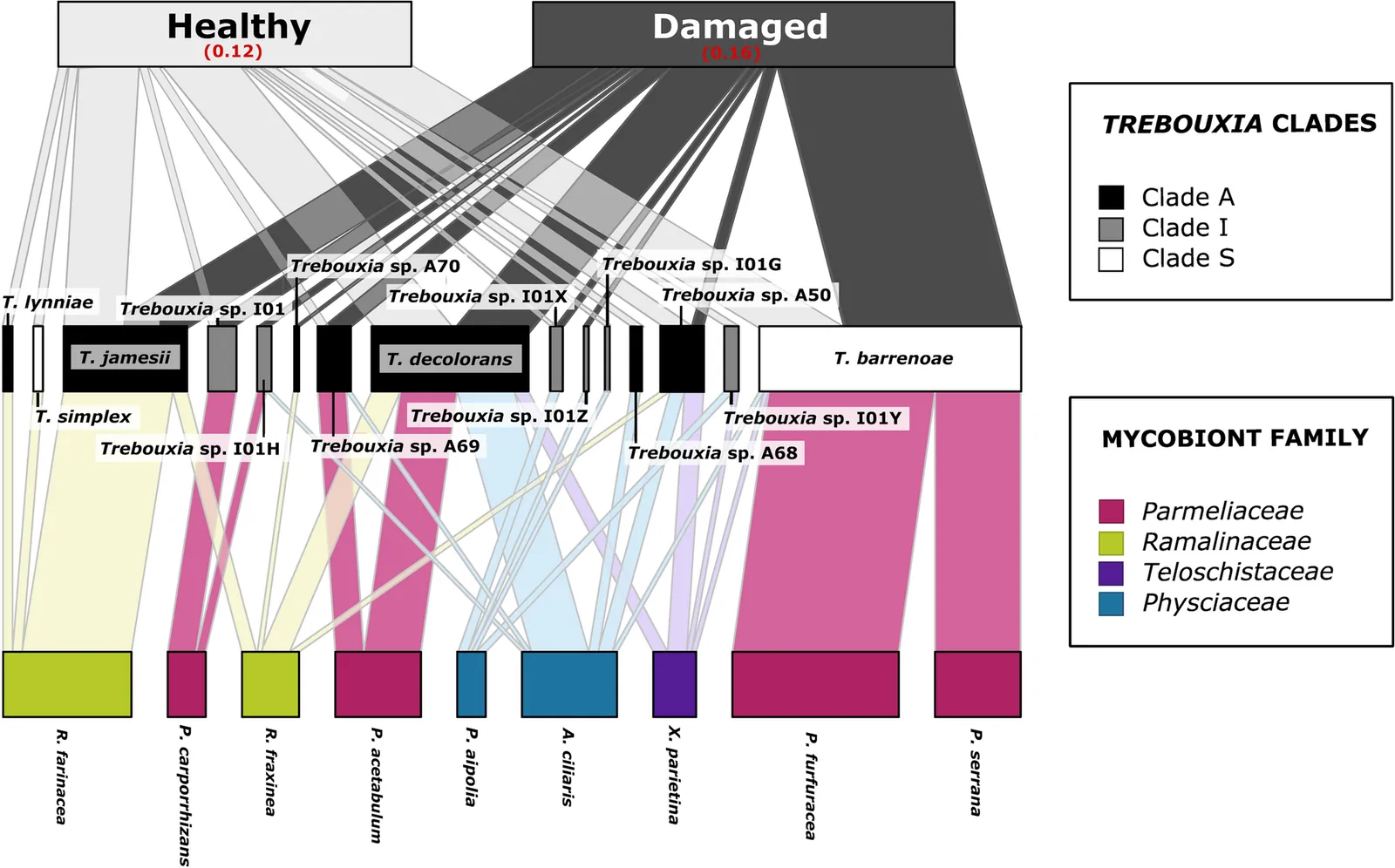
Interaction network between phycobiont lineages and the thallus health status. Mycobiont species are shown in the lower-most boxes. Boxes representing mycobionts are colour-coded based on fungal families, whereas colours of phycobiont boxes (central panel) follow a grayscale scheme, with white representing members of *Trebouxia* clade S, black representing clade A, and grey representing clade I. Link widths are proportional to the number of specimens. For interpretation purposes, links connecting myco- and phycobiont species are highlighted regarding the mycobiont family colour. The *d’* specialization index for the thallus health status is highlighted in red

An interaction network illustrating links between thallus damage status, phycobiont lineages, and mycobiont partners is presented in Fig. 2. Healthy lichen thalli (n = 70) displayed greater phycobiont diversity, encompassing 12 *Trebouxia* species-level lineages in comparison with 10 lineages detected in damaged thalli (n = 83). In both conditions, *Trebouxia* species from clades A, I, and S were represented. Several phycobiont partners occurred exclusively in healthy thalli (e.g., *T. lynniae*, *T. simplex*, and *Trebouxia* sp. A68, I01G, and I01Y), while others were restricted to damaged thalli (e.g., *Trebouxia* sp. A70, I01H, and I01Z). Most species-level phycobionts (n = 8), however, occurred in both categories. Notably, the three most widespread and abundant phycobionts in the dataset — *T. decolorans*, *T. barrenoae*, and *T. jamesii* — were associated with multiple mycobiont species across healthy and damaged thalli. Specialization values (*d*’) with respect to thallus health status were generally low, with *d’ = 0.12* for healthy lichens and *d’ = 0.16* for damaged ones (Fig. 2). No clear signal of phycobiont selectivity or strong health-dependent specialization was observed. From the phycobiont perspective, all species-level lineages exhibited specialization values below 0.20 (Supplementary Table S3).

To further explore these patterns, lichen samples were also analysed according to discrete DI levels from 1 to 5. The highest diversity was recorded at DI = 1 (12 phycobiont lineages, *n* = 70). At DI = 2, 5 phycobiont lineages were reported (*n* = 32), DI = 3 (7, *n* = 25), DI = 4 (6, *n* = 17), and DI = 5 (4 lineages, *n* = 9) (Supplementary Fig. S6). At that finer resolution, the most frequent phycobiont in the assembled dataset, *Trebouxia barrenoae* and *T. decolorans*, were present across all five DI levels. Moreover, *T. jamesii*, the third most abundant phycobiont, was observed to be involved in symbiosis with lichens from all damage levels except DI = 5. Specialization (*d’*) values calculated for all DI levels were generally low, ranging from 0.0793 in DI = 3 to 0.2081 in DI = 5 (Supplementary Fig. S6). Statistical analyses revealed no significant differences in *d’* values between the different DI levels (Kruskal-Wallis test p-value = 0.406).

### Phycobiota Taxonomic Composition

Illumina sequencing yielded a total of 1,878,415 reads that represented an average of 10,099 reads (minimum = 503, maximum = 119,911, median = 8,764.5) per sample. All sequenced samples revealed the presence of more than one microalgal species within the lichen thallus. The dataset encompassed 246 swarms, assigned to 107 microalgal lineages (ranging from 2 to 47 per sample, mean = 22), of which only 44 represented formally described species. Within this diverse microalgal assemblage, the genus *Trebouxia* clearly dominated the lichen phycobiota.

#### Dominance of *Trebouxia* Lineages Within the Lichen Phycobiota

A total of 92.35% of all reads (*n* = 1,734,619) were taxonomically assigned to the genus *Trebouxia*, encompassing 74 swarms that were assigned to 51 lineages. Of these, seven species were assigned to clade A, three to clade I, and three to clade S. We also found 34 previously undescribed *Trebouxia* lineages reported by earlier studies (https://trebouxia.net; see Supplementary Table S4 for taxonomic adscription of lineages). Furthermore, lineages *Trebouxia* sp. A68, A69, and A70 (see Section 3.1.1) were also detected, together with a newly identified lineage, *Trebouxia* sp. S34. This lineage was identified from a partial ITS sequence obtained through metabarcoding. Consequently, and following the criteria established by the Trebouxia Web Portal, partial ITS sequences cannot be considered reference sequences. Therefore, after comparison with sequences available in GenBank, sequence PP337168 was selected as the representative reference sequence for lineage S34. Finally, four previously unreported *Trebouxia* taxa—three in clade A and one in clade I—were also detected at very low abundances, never exceeding 11% of reads even in the thalli where they were most frequent. Complete ITS sequences could not be recovered for any of these taxa in the GenBank; accordingly, we have not assigned formal codes and provisionally refer to them as *Trebouxia* sp. 1, sp. 2, sp. 3, and sp. 4.

#### Diverse but Low-Abundance Assemblage of Non-*Trebouxia* Microalgae

**Fig. 3:**
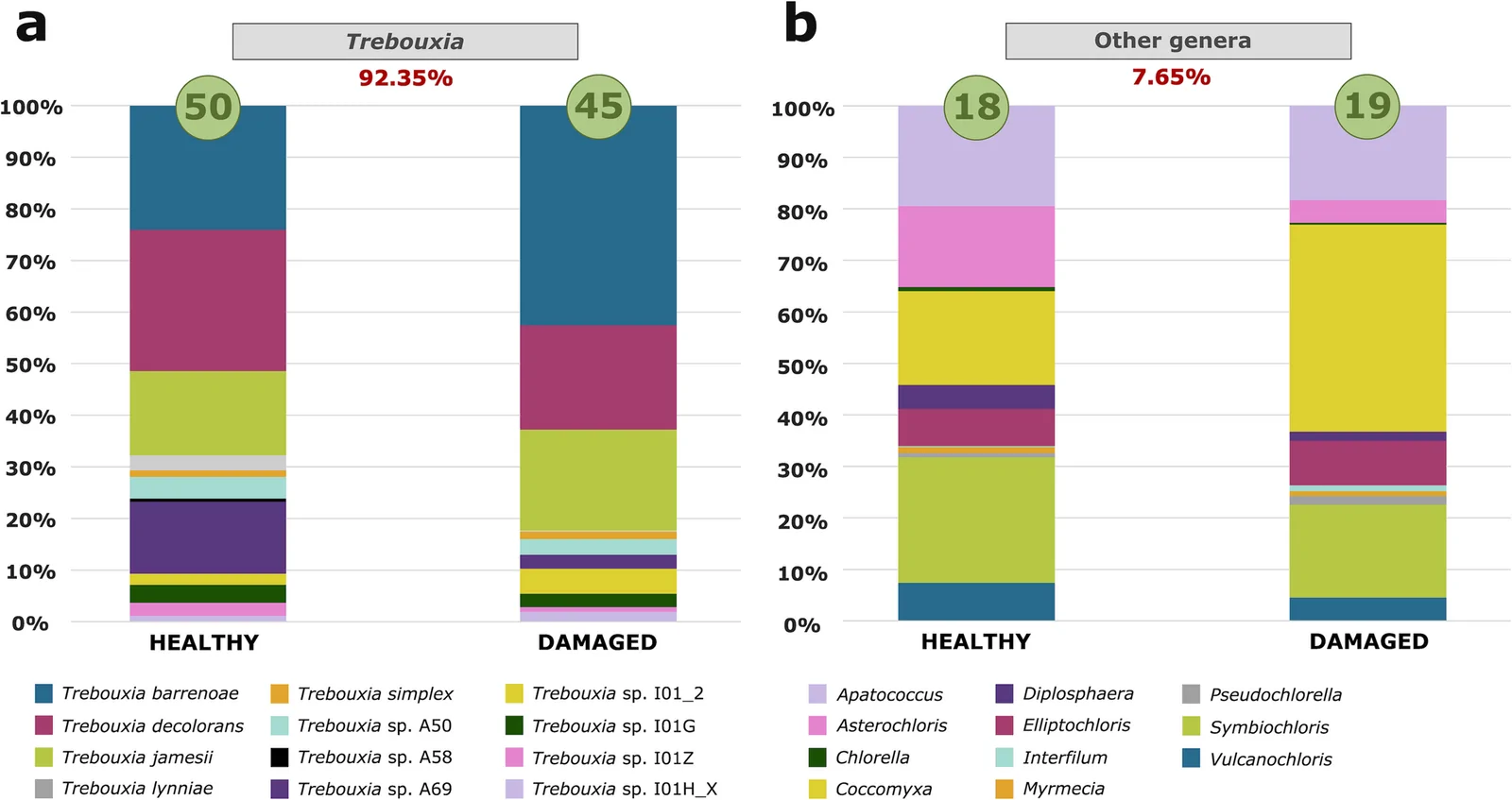
**(a)** Relative abundance graph of *Trebouxia* spp. across all samples (*n* = 186) categorized by thallus health status. For clarity, only species-level lineages representing more than 0.5% of the total abundance of swarms were shown (*n* = 12 for healthy lichens and *n* = 10 for damaged lichens). **(b)** Relative abundance graph of non-*Trebouxia* genera across all samples (*n* = 186) found in each locality. Only genera representing more than 0.5% of the total abundance of swarms were displayed (*n* = 11 for healthy and damaged lichens). The total number of detected *Trebouxia* microalgae is highlighted in a green circle. The percentage of reads corresponding to *Trebouxia* and other genera is highlighted in red

In addition to *Trebouxia*, a diverse assemblage of other microalgal genera was detected at much lower abundances. The remaining 7.65% of reads (*n* = 143,796) corresponded to 19 microalgal genera detected in much lower proportions. These included, in descending order of relative abundance: *Coccomyxa* (5 lineages, 33.56% of the total non-*Trebouxia* reads), *Symbiochloris* (8 lineages, 19.78%), *Apatococcus* (7 lineages, 18.51%), *Elliptochloris* (6 lineages, 8.23%), *Asterochloris* (10 lineages, 7.7%), *Vulcanochloris* (1 species, 5.36%), and *Diplosphaera* (4 lineages, 2.52%). Genera with a relative abundance below 1.5% included: *Pseudochlorella*, *Myrmecia*, *Interfilum*, *Chlorella*, *Pseudostichococcus*, *Chloroidium*, *Tritostichococcus*, *Leptosira*, *Lobosphaera*, *Deuterostichococcus*, *Gloeocystis*, and *Protostichococcus* (see Supplementary Table S4 for taxonomic classification at the species level). Overall, 172 swarms belonging to non-*Trebouxia* genera were assigned to 56 species-level lineages. A graphical representation of the relative abundances of *Trebouxia* species and less prevalent genera is shown in Fig. 3.

#### Phycobiota Composition Responds to Increasing Thallus Damage

Among the 186 sequenced thalli using Illumina, the most abundant microalga—defined as the taxon with the highest number of sequence reads— on each thallus corresponded to its primary phycobiont identified by Sanger sequencing in 166 samples (89.75%). In 70% of samples, this dominant microalga accounted for more than 80% of total reads within each thallus. Among the 51 *Trebouxia* species-level lineages, only 12 exceeded 0.5% of the total diversity, based on the total number of reads. Notably, over 70% of all reads corresponded to the three dominant phycobionts: *Trebouxia barrenoae*, *T. decolorans*, and *T. jamesii*. The remaining 30% was distributed among other *Trebouxia* species with varying proportions across damage levels. Most *Trebouxia* taxa were shared between healthy and damaged lichen thalli; however, *Trebouxia* sp. A13, A53, A56, A58, A68, and *Trebouxia* sp. 1 were exclusively detected in healthy thalli, whereas *Trebouxia* sp. A70 was found solely in damaged ones (DI = 2, 3, and 5). A decreasing trend in species diversity was observed with increasing damage. Healthy lichen thalli (DI = 1, *n* = 81) exhibited the highest diversity, with 50 microalgal species, whereas damaged thalli (*n* = 105) hosted 45 species-level lineages: 40 lineages at DI = 2 (*n* = 38), 37 at DI = 3 (*n* = 35), 34 at DI = 4 (*n* = 22), and 27 at DI = 5 (*n* = 10) (Supplementary Fig. S7).

Regarding non-*Trebouxia* genera, the number of reads in damaged thalli (*n* = 101,137) was more than double that recorded in healthy lichens (*n* = 42,457). In terms of diversity, all 19 non-*Trebouxia* genera were detected in damaged thalli, whereas 18 genera —except for *Gloeocystis*— were found in healthy thalli. Most genera were shared between healthy and damaged thalli and occurred in similar proportions; however, the genus *Asterochloris* was predominantly found in healthy thalli, with 6,651 reads compared to 4,416 reads in damaged samples. In contrast, the genus *Coccomyxa* was more abundant in damaged thalli, representing 40,480 reads *versus* 7,713 reads in healthy thalli.

### Alpha Diversity

Rarefaction curves, generated according to thallus health status (i.e., healthy *versus* damaged), and across DI categories (Supplementary Fig. S8, S9) showed clear saturation plateaus, indicating that sequencing depth was sufficient and the number of undetected swarms was likely minimal.

**Fig. 4:**
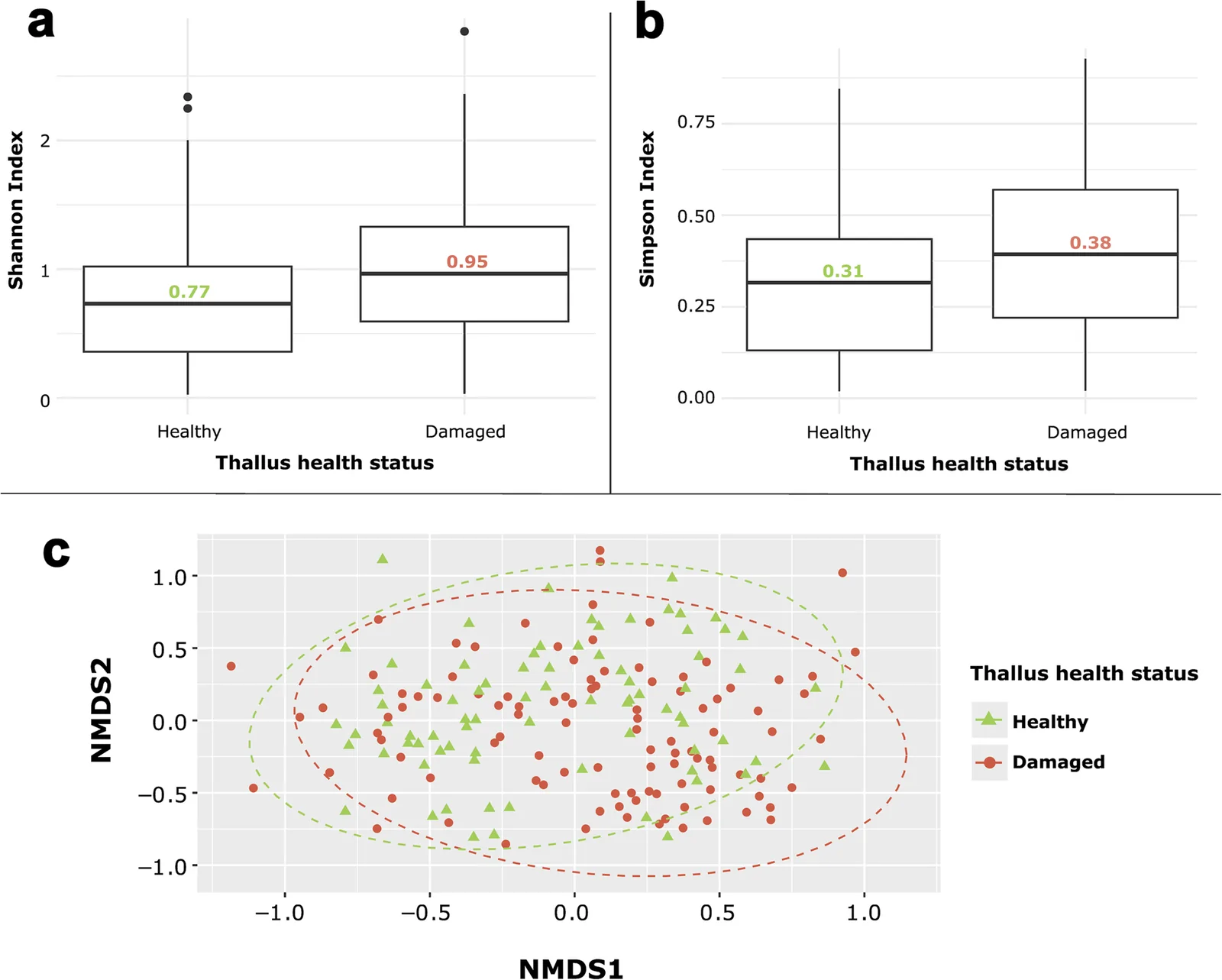
**(a)**Shannon and **(b)** Simpson diversity indices of the lichen phycobiota relative to thallus health status. Boxplots show medians, interquartile ranges, and numeric median values. **(c)** NMDS ordination plot of microalgal communities according to thallus health status. Different colours and shapes represent health status. Ellipsoid hulls encircle the health status (also indicated by colours)

Diversity within the lichen phycobiota was assessed using Shannon and Simpson indices, calculated for both thallus health status (Fig. 4) and the five DI categories (Supplementary Fig. S10). The Shannon index revealed relatively high median diversity values of 0.77 for healthy thalli and 0.95 for damaged ones. Kruskal-Wallis tests indicated statistically significant differences among categories (*p* = 0.0387). Similarly, the Simpson index showed consistent medians between groups (0.31 for healthy *versus* 0.38 for damaged thalli), with significant differences also detected (Kruskal–Wallis test *p* = 0.0462) among categories. Across DI categories, diversity was lowest at DI = 1 (median = 0.77) and increased towards intermediate damage levels (DI = 2, 0.94, DI = 3, 0.95, DI = 4, 0.97), but decreased again at DI = 5 (median = 0.85) (Supplementary Fig. S10).

###  Beta Diversity

Non-metric multidimensional scaling (NMDS) ordination (Fig. 4) was calculated to further explore differences in community composition among damage categories. Results revealed a high degree of overlap between phycobiotas in the ordination space across the different thallus health categories. However, PERMANOVA tests indicated a statistically significant but weak differentiation among damage categories (*p* = 0.019), with the explained variance being low (R² = 1.43%). No well-defined clusters were observed. Furthermore, permutation tests for homogeneity of dispersion (*p* = 0.336) confirmed that the observed differences were not driven by within-group heterogeneity.

## Discussion

In this study, we investigated whether the visible damage of lichen thalli in nine epiphytic macrolichens was associated with shifts in the identity of the primary phycobiont and changes in the diversity and/or structure of the accessory microalgal community. Our results revealed that all thalli hosted multiple microalgal lineages. However, the overall diversity of the phycobiota was strongly dominated by a few *Trebouxia* species, and morphological thallus damage exerted only a limited influence on its structure.

Sanger sequencing identified 16 species-level lineages spanning *Trebouxia* clades A, S, and I [5, 8]. Among them, *T. barrenoae* was the predominant, followed by *T. decolorans* and *T. jamesii*, while the remaining taxa occurred at lower frequencies. Comparable patterns were observed in other Mediterranean holm oak-dominated forests [43], whereas higher *Trebouxia* diversity occured in the humid, temperate Białowieża forest [41]. The absence of clades C and D in our study is not unexpected for these latitudes, as clade C is predominantly tropical [125] and clade D is typically found in boreal/northern areas [126]. Beyond bark-dwelling communities, lichen symbioses on soils and rocks often harbor a broader range of photobiont genera, frequently extending beyond *Trebouxia*. For example, *Asterochloris* dominates terricolous *Cladonia* lichens [37], while saxicolous lichens associate with *Myrmecia* and *Asterochloris* in addition to *Trebouxia* [35, 36, 127]. Collectively, these findings highlight the need for broader surveys to uncover the full extent of lichen-associated photobiont diversity in the entire investigated forest community.

Furthermore, Illumina sequencing revealed a substantial expansion of the phycobiont diversity detected by Sanger sequencing, uncovering multiple *Trebouxia* lineages in all analyzed thalli. This result aligns with accumulating evidence that lichens host more complex phycobiont assemblages than traditionally assumed, a pattern that early Sanger-based studies could only partially detect due to sensitivity limitations [15, 25, 128]. Remarkably, 92% of total reads belonged to *Trebouxia*, suggesting strong host-driven selectivity for this genus. Similar increases in detected phycobiont diversity have been documented in other lichen species, such as *R. farinacea* [44, 129] and *Xanthoria parietina*, where whole-genome analyses uncovered previously unrecognized lineages [130]. Metagenomic reconstructions of lichen hologenomes further support the view that individual thalli host complex symbiotic assemblages, revealing multiple phycobiont strains together with diverse bacterial and fungal counterparts [47, 64]. Additional studies further support that phycobiont plurality within thalli is a common phenomenon [48, 129, 131–133], indicating that our observed patterns represent a widespread characteristic of lichen symbioses.

Despite the high number of microalgal lineages detected, most occurred at very low abundances, with only a few representing more than 0.5% of the total reads per thallus. In contrast, the dominant phycobiont accounted for more than 80% of the total diversity in most thalli and matched the main species previously detected by Sanger. These results suggest that lichen symbioses are strongly dominated by a single phycobiont lineage, with co-occurring microalgae playing a marginal role in terms of relative abundance. The ratios of co-existing phycobiont within individual thalli in various studies exhibit a similar trend, since most thalli indeed contain a single, clearly predominant species [133–138]. The functional role of these low-abundance microalgae remains unresolved: some may represent neutral passengers without significant contribution to the symbiosis, whereas others might act as potential reservoirs of symbiotic partners, facilitating partner switching under changing environmental conditions [93, 129, 135]. Future functional studies integrating physiology, transcriptomics, and environmental stress experiments are needed to clarify their ecological significance.

To explore the adaptive role of phycobiont plurality under variable environmental conditions, a few studies addressing the physiology of coexisting species within a single lichen thallus have shown that different partners respond differently to stress conditions [15, 44, 139], suggesting that, while the primary phycobiont largely determines the overall stress response [140], the presence of multiple lineages that combine their physiological properties may provide an advantage for colonizing new habitats [141] and may be essential for survival in polluted areas or changing environments. Our results considering Sanger data did not provide evidence for a clear relationship between phycobiont identity and thallus health status. Most *Trebouxia* lineages in our dataset were found associated with both healthy and damaged lichens, suggesting no specific association of a particular phycobiont lineage with either damage status. Nevertheless, phycobiont diversity in healthy thalli was greater than the diversity found in damaged thalli. However, this differential stress tolerance or relative resilience of phycobionts should be interpreted cautiously, due to unequal representation of lichen specimens among the different damage levels.

When examined at a higher resolution using Illumina sequencing, more complex patterns emerge: damaged thalli hosted fewer phycobiont taxa overall, but displayed higher alpha diversity than healthy thalli. This paradox likely reflects a more even distribution of microalgae abundances within damaged thalli, where dominance weakens and rarer lineages become relatively more abundant. Beta diversity analyses further supported this view, showing a high degree of community overlap across health states, with only weak but statistically significant differentiation among damage categories. Notably, non-*Trebouxia* genera were consistently present across all levels of damage, revealing that visual thallus damage has a limited structuring effect on overall microalgal composition. Nevertheless, the genus *Asterochloris* was predominantly associated with healthy thalli, whereas *Coccomyxa* increased in relative abundance in damaged thalli. Previous studies revealed that *Asterochloris* spp. are highly sensitive to environmental stress [142], and thus lichens colonizing polluted habitats often replace *Asterochloris* with more tolerant species of *Trebouxia* [143, 144]. In contrast, *Coccomyxa* has been reported to maintain its metabolic activity under acidic stress, reflecting its resilience in degraded environments [16, 145]. Collectively, these findings suggest that environmental stress, perhaps derived from air pollution, does not induce a categorical microalgal shift, but rather promotes subtle restructurings towards more balanced communities. Such results are in accordance with previous observations indicating that hosting multiple phycobionts appears to perform better in air-polluted environments [143], likely because stress conditions weaken dominant partners and allow low-abundance taxa to increase their presence, thereby restructuring communities towards greater photosynthetic balance.

Recent debates on the definition of lichens have prompted some authors to re-conceptualize them as ecosystems [3, 146]. Under this perspective, lichens encompass a diverse set of accessory biological communities (e.g., epiphytic and endophytic bacteria, yeasts, cyanobacteria, lichenicolous fungi, etc.), and thus their dynamics can be examined in light of the Intermediate Disturbance Hypothesis (IDH; [88, 147]), which states that biodiversity is maximized at intermediate levels of perturbation, where both disturbance-sensitive and disturbance-tolerant species can coexist. Here we found that the microalgal communities’ diversity peaks at intermediate levels of lichen damage (DI = 2–4), and declines at the extremes. No previous study has examined the lichen phycobiota under the framework of the IDH; however, some evidence from lichen richness studies illustrates the ongoing debate regarding the generality of this hypothesis. For example, Pastore et al. [148] reported that the diversity of a saxicolous lichen community reached its highest level after 32 years of intermediate disturbance, and they also suggested that the coexistence of lichens in the system was regulated by differences in their persistence and growth rates. Similarly, studies on epiphytic macrolichens in boreal forests have shown that lichen richness can be highest in forest stands of intermediate age after disturbance, likely because both early-colonizing species and later-successional species can coexist under these conditions [149]. By contrast, Boggess et al. [150] analysed the impact of human disturbance in eastern North American forests using lichen species richness and found a consistent negative relationship between disturbance intensity and species diversity, concluding that biodiversity declines monotonically with disturbance. Evidence for IDH-like processes has been observed at the community level, but considering multiple ecological scales may help reconcile contrasting patterns. Within a single locality, thalli of the same lichen species can be dominated by different *Trebouxia* lineages [41, 129]. We hypothesize that at intermediate levels of damage or stress, weakened environmental filtering and reduced competitive exclusion by the primary phycobiont facilitate the coexistence of alternative lineages, resulting in divergent dominance among neighboring thalli. At the thallus (individual) scale, some specimens lack a clearly dominant algal partner and instead harbor several algal taxa at comparable relative abundances—often complicating Sanger sequencing [25, 44, 151]—a within-thallus co-dominance that is consistent with IDH expectations. However, while our results provide preliminary support for IDH patterns in lichen-associated microalgal communities, they should be interpreted cautiously, as sampling biases and methodological limitations prevent us from a definitive evaluation of this hypothesis.

Despite advances in molecular approaches, significant challenges remain in characterizing symbiotic *Trebouxia*: species delimitation within clades, particularly clade I, continues to be problematic, and the boundaries among formally described species are often unresolved. These limitations hinder new species delimitation within the clade and underscore the need for integrative approaches combining multiple molecular markers and incorporating morphological and ecological data [39]. Future studies should also examine other microbial components of the lichen holobiont, including fungi and bacteria, to assess their diversity in Mediterranean epiphytic lichens and responses to thallus damage. Furthermore, although the responses of lichens to pollution and environmental stress have been relatively more studied (e.g. [39, 150]), the direct impacts on the photosynthetic partner remain poorly understood, suggesting that shifts in microalgal assemblages may mediate the overall sensitivity of lichens to disturbance. Given their ecological significance, sensitivity, and multifunctional roles, lichens remain powerful bioindicators of environmental change, and our findings reinforce their value for assessing the impacts of disturbance and stress in forest systems.

## Conclusion

This work provides the first comprehensive evaluation of the diversity of microalgae inhabiting lichen thalli under the impact of environmental stress, offering valuable insights into their identity and community structure in Mediterranean forests. Using Sanger and next-generation sequencing techniques, we found that lichen phycobiota in our dataset are consistently dominated by a few *Trebouxia* lineages, with additional microalgal taxa occurring at very low abundances. Visible thallus damage had minor effects on the identity of the primary phycobiont, but was associated with subtle restructuring of accessory microalgal communities. Phycobiont diversity peaked at intermediate levels of thallus damage, consistent with the idea that microalgal community evenness is modulated by disturbance rather than a categorical shift in symbiotic composition. These findings suggest that a dominant phycobiont maintains the structural core of the symbiosis, while accessory microalgae may enhance resilience under adverse environmental conditions. To fully resolve how morphological damage in lichen thalli influences their internal symbiotic architecture, a deeper understanding of the diversity and roles of all components of the lichen holobiont is essential.

## Supplementary Information

Below is the link to the electronic supplementary material.

Supplementary material 1 (XLS 221 KB)

Supplementary material 2 (DOCX 14.5 MB)

Supplementary material 3 (PNG 1.79 MB)

Supplementary material 4 (PNG 1.96 MB)

Supplementary material 5 (PNG 2.80 MB)

Supplementary material 6 (PNG 329 KB)

Supplementary material 7 (PNG 3.05 MB)

Supplementary material 8 (PNG 889 KB)

Supplementary material 9 (PNG 1.06 MB)

Supplementary material 10 (PNG 1.33 MB)

Supplementary material 11 (PDF 8.77 MB)

Supplementary material 12 (PNG 56.7 MB)

## Data Availability

All data supporting the findings of this study are available within the paper and its Supplementary Material.
