# Supplementary material for: The Impact of Visible Symptoms of Thallus Damage on the Phycobiota of Mediterranean Epiphytic Lichens
Source: Microb Ecol. 2026 Jun 9;89:158. doi: 10.1007/s00248-026-02794-3 (PMC13473299; doi:10.1007/s00248-026-02794-3)
Supplement: Supplementary file 1 [file 248_2026_2794_MOESM11_ESM.pdf]

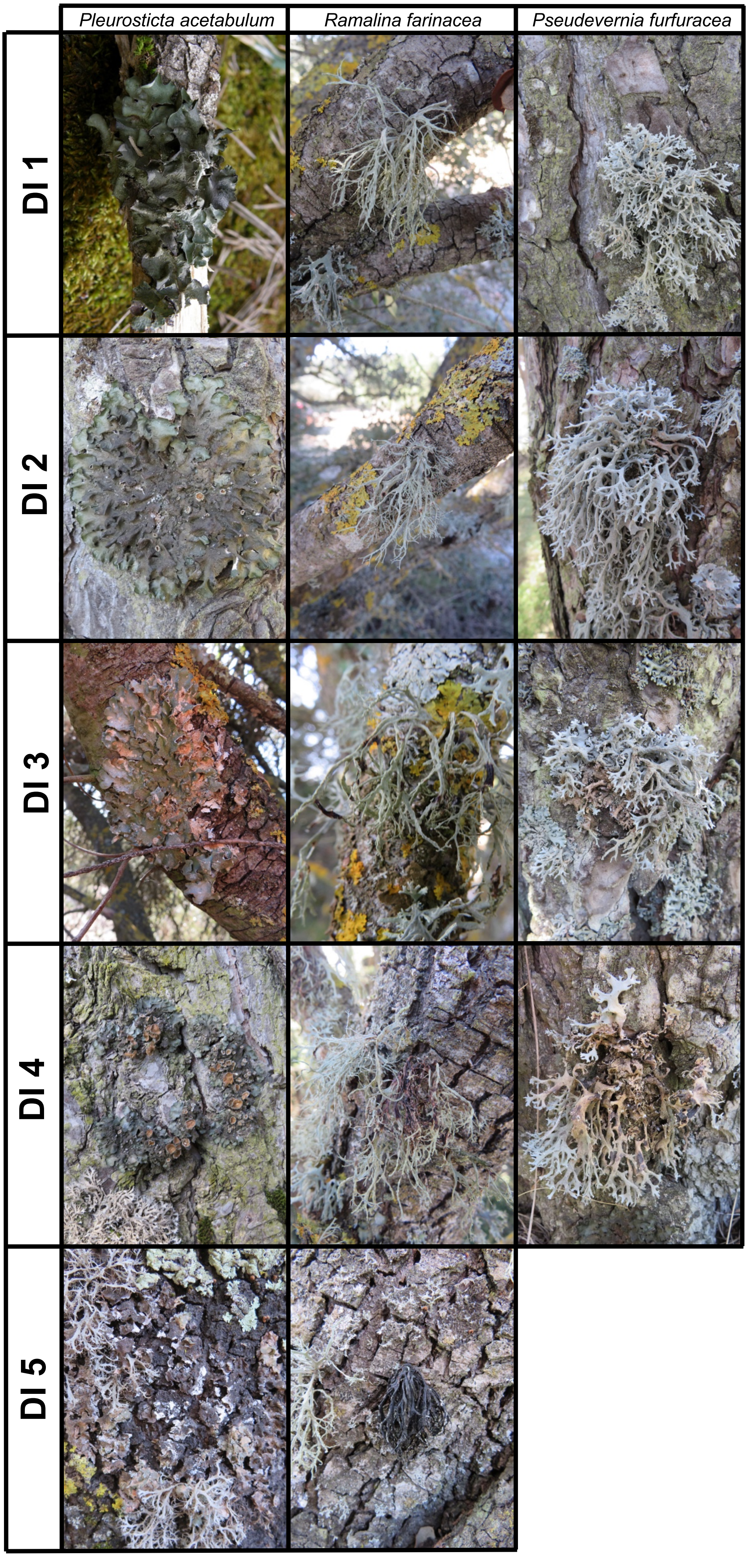
Pleurosticta acetabulum
Ramalina farinacea
Pseudevernia furfuracea
DI 1
DI 2
DI 3
DI 4
DI 5
