# Supplementary material for: The Impact of Visible Symptoms of Thallus Damage on the Phycobiota of Mediterranean Epiphytic Lichens
Source: Microb Ecol. 2026 Jun 9;89:158. doi: 10.1007/s00248-026-02794-3 (PMC13473299; doi:10.1007/s00248-026-02794-3)
Supplement: Supplementary file 2 [file 248_2026_2794_MOESM2_ESM.docx]

**The impact of visible symptoms of thallus damage** **on the phycobiota of Mediterranean epiphytic lichens**

Tamara Pazos^1^, Patricia Moya^1^, Salvador Chiva^1^, Pavel Škaloud^2^, Veronika Kantnerová^2^, Eva Barreno^1^ & Isaac Garrido-Benavent^3^.

1. Instituto Cavanilles de Biodiversidad y Biología Evolutiva (ICBiBE)– Botánica, Universitat de València, C/Dr. Moliner, 50, E-46100 Burjassot, València, Spain, *Corresponding author e–mail: tamara.pazos@uv.es
2. Department of Botany, Faculty of Science, Charles University, Benátská 2, Prague, 12800, Czech Republic
3. Departament de Botànica i Geologia, Universitat de València, C/Dr. Moliner, 50, E-46100 Burjassot, València, Spain

**SUPPLEMENTARY FIGURE**

**Supplementary Figure S1.** Field-based visual examples of Damage Index (DI) categories for selected macrolichen species. DI values (1–5) reflect increasing severity of thallus damage, from no visible symptoms (1) to necrosis or dead thalli (5).

**Supplementary Figure S2.** Phylogram inferred with MrBayes using a phycobiont ITS sequence dataset that depicts phylogenetic relationships among algal partners of the selected species. Labels on different-coloured boxes correspond with species-level lineages within Trebouxia clade A as proposed by Muggia et al. ([2020](https://www.cambridge.org/core/journals/lichenologist/article/punctelia-borreri-and-p-subrudecta-parmeliaceae-associate-with-a-partially-overlapping-pool-of-trebouxia-gelatinosa-lineages/E1C68C8B4A746E7506D3A3F4C892EFFC#ref48)). Posterior probabilities (PP) are indicated with different coloured branches. Data on the phorophyte (*Pinus* or *Quercus*) and the mycobiont species associated with each newly sequenced specimen is provided as symbols to the right of the specimen's label.

**Supplementary Figure S3.** Phylogram inferred with MrBayes using a phycobiont ITS sequence dataset that depicts phylogenetic relationships among algal partners of the selected species. Labels on different-coloured boxes correspond with species-level lineages within Trebouxia clade S as proposed by Muggia et al. ([2020](https://www.cambridge.org/core/journals/lichenologist/article/punctelia-borreri-and-p-subrudecta-parmeliaceae-associate-with-a-partially-overlapping-pool-of-trebouxia-gelatinosa-lineages/E1C68C8B4A746E7506D3A3F4C892EFFC#ref48)). Posterior probabilities (PP) are indicated with different coloured branches. Data on the phorophyte (*Pinus* or *Quercus*) and the mycobiont species associated with each newly sequenced specimen is provided as symbols to the right of the specimen's label.

**Supplementary Figure S4.** Phylogram inferred with MrBayes using a phycobiont ITS sequence dataset that depicts phylogenetic relationships among algal partners of the selected species. Labels on different-coloured boxes correspond with species-level lineages within Trebouxia clade I as proposed by Muggia et al. ([2020](https://www.cambridge.org/core/journals/lichenologist/article/punctelia-borreri-and-p-subrudecta-parmeliaceae-associate-with-a-partially-overlapping-pool-of-trebouxia-gelatinosa-lineages/E1C68C8B4A746E7506D3A3F4C892EFFC#ref48)). Posterior probabilities (PP) are indicated with different coloured branches. Data on the phorophyte (*Pinus* or *Quercus*) and the mycobiont species associated with each newly sequenced specimen is provided as symbols to the right of the specimen's label.

**Supplementary Figure S5.**

MrBayes phylogeny of the mycobiont

ITS sequence dataset that depicts phylogenetic

relationships among mycobiont partners of

the selected species. Thickened branches

indicate bootstrap support (BS, RAxML) ≥ 70%

and/or posterior probabilities (PP, MrBayes)

≥ 0.95. Data regarding the phorophyte

(*Pinus* or *Quercus*) where the specimen was

growing is provided as symbols to the right of

the specimen's label.

**Supplementary Figure S6.** Interaction network between phycobiont lineages and the registered DI. Mycobiont species are shown in the lower-most boxes. Boxes representing mycobionts are color-coded based on fungal families, whereas colours of phycobiont boxes (central panel) follow a grayscale scheme, with white representing members of *Trebouxia* clade S, black representing clade A, and grey representing clade I. Link widths are proportional to the number of specimens. For interpretation purposes, links connecting myco- and phycobiont species are highlighted regarding the mycobiont family colour. The *d’* specialization index for the DI is highlighted in red.

**Supplementary Figure S7.** A. Relative abundance graph of *Trebouxia* spp. across all samples (n=186) categorized by the Damage Index (DI). For clarity, only species-level lineages representing more than 0.5% of the total abundance of swarms were shown. B. Relative abundance graph of non-*Trebouxia* genera across all samples (n=186) found in each locality. Only genera representing more than 0.5% of the total abundance of swarms were displayed (n = 12). The total number of detected *Trebouxia* microalgae is highlighted in a green circle.

**Supplementary Figure S8.** Rarefaction curves showing swarm richness as a function of sequencing depth (number of reads per sample) for each sample categorized according to the thallus health status. Each curve represents an individual thallus.

**Supplementary Figure S9.** Rarefaction curves showing swarm richness as a function of sequencing depth (number of reads per sample) for each sample categorized according to the Damage Index (DI). Each curve represents an individual thallus.

**Supplementary Figure S10.** (A) Shannon and (B) Simpson diversity indices of the lichen phycobiota relative to the Damage Index (DI) five categories. Boxplots show medians, interquartile ranges, and numeric median values.
